# Exploring key genes associated with neutrophil function and neutrophil extracellular traps in heart failure: a comprehensive analysis of single-cell and bulk sequencing data

**DOI:** 10.3389/fcell.2023.1258959

**Published:** 2023-10-24

**Authors:** Xudong Li, Changhao Xu, Qiaoqiao Li, Qingxiang Shen, Long Zeng

**Affiliations:** ^1^ State Key Laboratory of Organ Failure Research, Department of Cardiology, Nanfang Hospital, Southern Medical University, Guangzhou, China; ^2^ Department of Cardiology, The First Affiliated Hospital of Nanjing Medical University, Nanjing, China; ^3^ Department of Cardiology, The Second Affiliated Hospital of Chongqing Medical University, Chongqing, China; ^4^ Department of Obstetrics and Gynecology, The Second Affiliated Hospital of the University of South China, University of South China, Hengyang, Hunan, China; ^5^ Department of Cardiology, Shangrao People’s Hospital, Shangrao, Jiangxi, China

**Keywords:** bulk RNA sequencing, heart failure, neutrophil, single-cell RNA sequencing, neutrophil extracellular traps (NET)

## Abstract

**Background:** Heart failure (HF) is a complex and heterogeneous manifestation of multiple cardiovascular diseases that usually occurs in the advanced stages of disease progression. The role of neutrophil extracellular traps (NETs) in the pathogenesis of HF remains to be explored.

**Methods:** Bioinformatics analysis was employed to investigate general and single-cell transcriptome sequencing data downloaded from the GEO datasets. Differentially expressed genes (DEGs) associated with NETs in HF patients and healthy controls were identified using transcriptome sequencing datasets and were subsequently subjected to functional enrichment analysis. To identify potential diagnostic biomarkers, the random forest algorithm (RF) and the least absolute shrinkage and selection operator (LASSO) were applied, followed by the construction of receiver operating characteristic (ROC) curves to assess accuracy. Additionally, single-cell transcriptome sequencing data analysis identified key immune cell subpopulations in TAC (transverse aortic constriction) mice potentially involved in NETs regulation. Cell-cell communication analysis and trajectory analysis was then performed on these key cell subpopulations.

**Results:** We identified thirteen differentially expressed genes (DEGs) associated with NET through differential analysis of transcriptome sequencing data from HF (heart failure) samples. Utilizing the Random Forest and Lasso algorithms, along with experimental validation, we successfully pinpointed four diagnostic markers (CXCR2, FCGR3B, VNN3, and FPR2) capable of predicting HF risk. Furthermore, our analysis of intercellular communication, leveraging single-cell sequencing data, highlighted macrophages and T cells as the immune cell subpopulations with the closest interactions with neutrophils. Pseudo-trajectory analysis sheds light on the differentiation states of distinct neutrophil subpopulations.

**Conclusion:** In this study, we conducted an in-depth investigation into the functions of neutrophil subpopulations that infiltrate cardiac tissue in TAC mice. Additionally, we identified four biomarkers (CXCR2, FCGR3B, VNN3, and FPR2) associated with NETs in HF. Our findings enhance the understanding of immunology in HF.

## Introduction

As the immune system’s first line of defense against infections, neutrophils play a critical role. They regulate the immune response through three mechanisms: phagocytosis, degranulation, and the release of NETs (Papayannopoulos, 2018). NETs are a significant component of the innate immune response. They entrap and eliminate pathogenic microorganisms, including viruses, bacteria, fungi, and protozoa, to prevent their wider spread *in vivo* (Carmona-Rivera et al., 2019). NETs are assembled from cytolytic and granular proteins, which are arranged on a dense chromatin scaffold. The formation of these structures results in the release of high concentrations of toxic proteins, which are lethal to entrapped microorganisms. The primary mode of NET release from neutrophils is through a cell death process known as NETosis. The multifaceted function of NETs in the immune system highlights their importance as an effective strategy against infectious diseases.

Recent research has shown that NETs play a crucial role in human immune responses, and their involvement in pathologies such as systemic lupus erythematosus (Kraaij et al., 2018; Papayannopoulos, 2018; Frangou et al., 2019a; Frangou et al., 2019b), rheumatoid arthritis (Khandpur et al., 2013; O'Neil et al., 2023; Sakkas et al., 2014; de Bont et al., 2020), and cystic fibrosis (Skopelja et al., 2016; Gray et al., 2018; Guerra et al., 2020; Morán et al., 2022) has been extensively studied. However, a growing body of literature also highlights their contribution to HF. Studies have found that cardiac pressure overload triggers NETosis, which can lead to a decrease in left ventricular ejection fraction (LVEF) in wild-type (WT) mice (Martinod et al., 2017). In Seipin/Bslc2 knockout mice, an Asian lean diabetic model, the formation of interstitial fibrosis associated with NETs exacerbates left ventricular sclerosis and further contributes to HF during its progression wang (Wang et al., 2019). While current research predominantly targets cardiomyocytes, fibroblasts, and other immune cell subsets as therapeutic targets, the potential contribution of non-resident immune cell subpopulations, such as neutrophils in cardiac tissue, to the development of HF remains poorly understood. Therefore, further investigation and development of the role of neutrophils in HF progression are necessary to better understand their potential therapeutic value.

The rapid development of Bulk RNA sequencing technology and single-cell sequencing technology has facilitated the discovery of new diagnostic and prognostic markers for diseases. In this study, we downloaded HF-related datasets from the GEO database and employed bioinformatics to screen for NET-related diagnostic markers in HF. Our analysis of intercellular communication, based on single-cell sequencing data, revealed that macrophages and T cells are the immune cell subpopulations with the most prominent interactions with neutrophils. Additionally, through pseudo-trajectory analysis, we gained insights into the differentiation status of various neutrophil clusters. These findings provide new insights into the role of NETs in HF and have significant implications for the development of targeted treatments and prevention strategies.

## Materials and methods

### Dataset and preprocessing

The RNA-seq datasets analyzed in this study were retrieved from the GEO database (https://www.ncbi.nlm.nih.gov/geo/) and consisted of 131 samples from 2 separate datasets. Of these, the GSE145154 dataset comprised 67 samples (52 HF cases and 15 healthy controls), while the GSE116250 dataset had 64 samples (50 HF cases and 14 healthy controls). The GSE145154 dataset was utilized as the training dataset, while the GSE116250 dataset was used as the validation dataset. It is worth noting that the 69 initial biomarkers of NETs included in this study were obtained from prior research studies (Zhang et al., 2022) (Supplementary Table S1).

### Identification of NETs-related differential genes

To identify differentially expressed genes (DEGs) associated with NETs, we conducted differential analysis on the training dataset samples utilizing the “Deseq2” package (version 1.38.2) (Anders and Huber, 2010). The established thresholds were set at *p*-value <0.05 and |logFC|>1. After intersecting DEGs with NETs genes, a final set of 13 NETs-related differential genes were obtained between HF and control samples.

### Identification of NETs-related diagnostic biomarkers

To pinpoint key NETs-related biomarkers, we harnessed the Random Forest (RF) algorithm and the LASSO regression model.

LASSO is a widely-used regression method for selecting variables to improve prediction accuracy, implemented through the “glmnet” R package (version 4.1), we selected the optimal λ value and removed genes that displayed partial collinearity to reduce potential bias. In contrast, the RF algorithm, a supervised classification method relying on decision trees, was executed using the “randomForest” R package (version 4.7). We evaluated error rates for tree counts ranging from 1 to 500 and determined the optimal number of trees by selecting the configuration with the lowest error rate. Furthermore, we gauged the feature importance scores for each gene, identifying candidate biomarkers as those with importance values exceeding 2 for subsequent analysis.

### Enrichment analysis

In our study, we employed the “clusterprofiler” R package (version 4.6.0) to conduct Gene Ontology (GO) and Kyoto Encyclopedia of Genes and Genomes (KEGG) pathway enrichment analyses (Yu et al., 2012). GO analysis is considered the gold standard for large-scale functional enrichment studies, as it covers various biological processes, molecular functions, and cellular components (The Gene Ontology Resource, 2019). Additionally, we utilized the KEGG database, which provides comprehensive information on genomes, biological pathways, diseases, and drugs (Kanehisa et al., 2023). Significant enrichment was defined as a critical *p*-value threshold of <0.05, and the results of the functional enrichment analysis are visually represented using bar charts.

### Single-cell data source and preprocessing

We processed the dataset GSE122930 using the “Seurat” R package (version 4.3.0) (Butler et al., 2018). Initial quality control involved the removal of cells based on the following criteria (Papayannopoulos, 2018): cells with fewer than 200 genes or more than 5,000 genes (UMI >0) (Carmona-Rivera et al., 2019), cells with more than 20,000 UMI, and (Frangou et al., 2019a) cells with over 12.5% mitochondrial UMI count. Subsequently, the data were log-normalized using default parameters. We selected the 894 most variable genes using the “FindVariableGenes” function and scaled the data using the “ScaleData” function to remove unnecessary sources of variation. Principal Component Analysis (PCA) was performed using the “RunPCA” function, and the number of principal components was determined visually using the “ElbowPlot” function. We constructed a shared nearest neighbor (SNN) plot for the first 15 principal components with the “FindNeighbors” function and clustered the cells using the “FindClusters” function, setting the “Resolution” parameter to 0.6. For visualization, we utilized the “RunUMAP” function to create UMAP plots. To identify marker genes in each cluster, we employed the “FindAllMarkers” function, setting the parameter “min.pct” to 0.2 and the “thresh.use” parameter to 0.2. Additionally, we used the “celltypist” to assist in cellular annotation (Domínguez Conde et al., 2022), followed by manual annotation for further refinement.

### Trajectory analysis of single cells

We used the CytoTRACE R package (version 0.3.3) to help predict the direction of cell differentiation (Gulati et al., 2020). For our single-cell trajectory analysis, we employed the R package Monocle2 (version 2.16.0) (Qiu et al., 2017). We initially identified clusters corresponding to cancer stem cells and epithelial cells, and subsequently loaded these clusters into the R environment. To facilitate the analysis, we created an object using the “newCellDataSet” function. Within the trajectory analysis, we harnessed the “FindVariableGenes” gene set to perform pseudo-temporal sorting of all cells within the target cell subpopulation. Next, we reduced the dimensions of the dataset using the “reduceDimension ()” function, utilizing the parameters “reduction_method = “DDRTree” and “max_components = 2.” For visualization purposes, we employed the “plot_cell_trajectory” function to generate a spanning tree of cells. Finally, we utilized the “differentialGeneTest” function to identify genes that exhibited significant changes over pseudotime [q-value <10^(-5)], and we visualized the expression changes of the top 100 genes over pseudotime using the “plot_pseudotime_heatmap” function.

### Cell-cell communication analysis

We utilized CellChat, a tool that quantitatively infers intercellular communication networks from scRNA-seq data (Jin et al., 2021). Based on a database of mouse ligand-receptor interactions and pattern recognition techniques, CellChat can detect intercellular communication at the pathway level and calculate the communication network of aggregated cells. Use default settings for all parameters.

### Transverse aortic constriction (TAC)

Male 8-week-old adult wild-type (WT) C57BL/6J mice were procured from Charles River Laboratory (Charles River, China). Briefly, after randomizing the mice into groups, consisting of three mice in the TAC group and three mice in the SHAM group, the mice were anesthetized using isoflurane and underwent a transthoracic thoracotomy. Following the exposure of the aortic arch, a suture was passed through the aortic arch positioned between the right innominate artery and the left common carotid artery. Subsequently, the aortic arch was ligated to a 27-gauge needle, and the needle was carefully withdrawn upon the completion of ligation. Mice in the sham-operated group underwent identical procedures but were not subjected to ligation. Following the surgical intervention, the mice were placed on a heating pad for recovery and closely monitored. Four weeks after either the sham or TAC surgery, the mice were anesthetized using an overdose of pentobarbital (100 mg/kg, Sigma-Aldrich), and their hearts were extracted through an open-chest procedure for subsequent molecular analysis.

### RNA isolation and real-time quantitative PCR (qRT-PCR)

Total RNA was extracted from cardiac tissue using Freezol reagent (Vazyme, R711), following the manufacturer’s instructions. Subsequently, qRT-PCR analysis was conducted on the QuantStudio™ 5 Real-Time PCR Detection System using ChamQ SYBR qPCR Master Mix (Low Rox Premixed) (Vazyme, Q331-02) and gene-specific primers. PCR analysis was performed on the QuantStudio™ 5 Real-Time PCR Detection System, with the following thermal cycling conditions: initial denaturation at 95°C for 1 min, followed by denaturation at 95°C for 10 s, annealing at 60°C for 30 s, and a total of 40 cycles. The relative expression levels of individual genes were quantified by the 2^(−ΔΔCt) method and normalized to the endogenous expression of glyceraldehyde-3-phosphate dehydrogenase (GAPDH). The sequences of the specific primers utilized for qRT-PCR in this study are provided in the below table.

**Table:** 

Gene name	Forword primer (5′→3′)	Reverse primer (5′→3′)
Mpo	AAT​ATG​GCA​CGC​CCA​ACA​AC	TCT​CCC​ACC​AAA​ACC​GAT​CAC
Vnn3	GCT​GTG​GGT​TCA​ATG​GAC​ACT	CTG​CCA​GCT​TGA​TTG​CAC​TCT
Cxcr2	GTA​ATT​CTG​GCC​CTG​CCC​AT	CAG​GAT​ACG​CAG​TAC​GAC​CC
Fcgr4	GAG​GTC​CAT​ATG​GGC​TGG​CTA	CTT​GCC​TTT​GCC​GTT​CTG​TAA
Fpr2	CAT​TTG​GTT​GGT​TCA​TGT​GCA​A	AAT​ACA​GCG​GTC​CAG​TGC​AAT
Gapdh	AGG​TCG​GTG​TGA​ACG​GAT​TTG	TGT​AGA​CCA​TGT​AGT​TGA​GGT​CA

### Statistical analysis

The validation of key gene expression differences between the experimental and control groups in both the training and validation datasets was conducted using the Wilcoxon rank sum test. Additionally, the qRT-PCR validation results were analyzed employing Student’s t-test. A significance threshold of *p* < 0.05 was applied to determine statistical significance. Data analysis and graph generation were performed using R software version 4.1.0 (http://www.R-project.org) and GraphPad Prism 8 (GraphPad Software, San Diego, CA, United States).

## Results

### Identification of differentially expressed NETs-related genes

**FIGURE 1:**
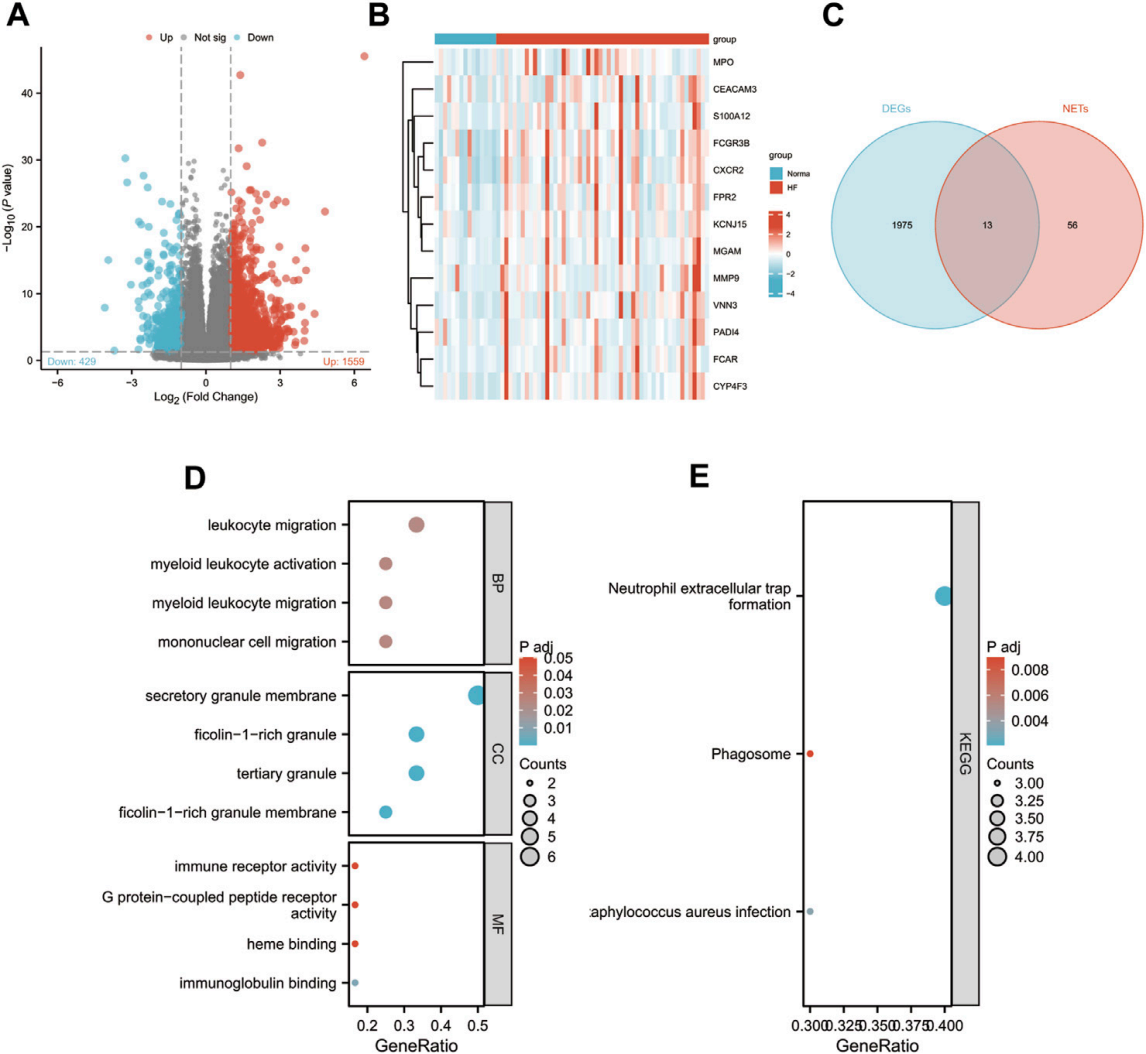
Identification of differentially expressed NETs-related genes. **(A)** Volcano plot of DEGs in GSE145154. **(B)** A Venn diagram of GSE145154 DEGs and NETs-related genes.**(C)** Expressions of differentially expressed NETs-related genes. **(D–E)** The enrichment pathway analysis of NETs-related DEGs.

To explore the role of NETs-related genes in HF pathogenesis, we conducted an analysis using the GSE145154 dataset, which consisted of 52 HF patient samples and 15 control samples. Following differential analysis between the two groups, we identified 1998 differentially expressed genes (DEGs), with 1,559 genes upregulated and 429 downregulated in the HF group compared to the control group (Figure 1A). We intersected the list of DEGs with known NETs-related genes, identifying a subset of 13 genes (Figure 1C; Supplementary Table S2). We also visualized the expression of these genes among different groups by heat map (Figure 1B). Through gene ontology (GO) enrichment analysis of the differentially expressed NETs-related genes, we found their involvement in leukocyte, myeloid leukocyte, and mononuclear cell migration. Additionally, the Kyoto Encyclopedia of Genes and Genomes (KEGG) pathway analysis suggested the activation of signaling pathways related to NET formation, phagosome, and *Staphylococcus aureus* infection (Figures 1D, E).

### Identification of neutrophil subpopulations associated with NETs

**FIGURE 2:**
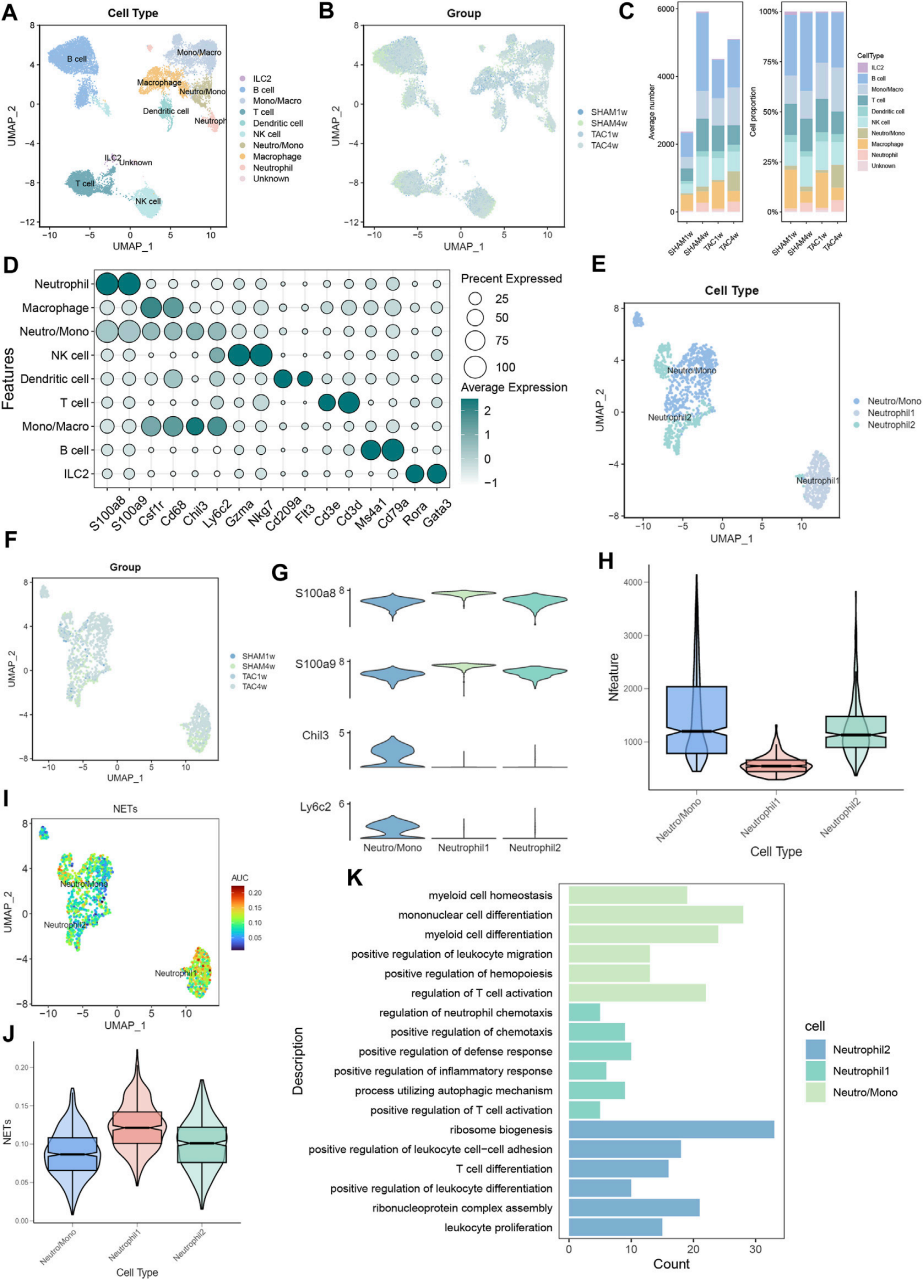
Identification of neutrophil subpopulations associated with NETs.**(A)** UMAP plots of 17,918 cells from 7 mouse heart samples.**(B)** UMAP plots showing the distribution of cells in SHAM1w, SHAM4w, TAC1w, and TAC4w heart samples.**(C)** Proportion of each cell subpopulation in different experimental groups.**(D)** Markers for different cell subpopulations.**(E)** UMAP plots of 1,451 neutrophils versus some monocytes from 7 mouse heart samples.**(F)** UMAP plots showing the distribution of neutrophils versus some monocytes in SHAM1w, SHAM4w, TAC1w, and TAC4w heart samples.**(G)** Violin plots showing markers for different neutrophil subpopulations.**(H)** Violin plots demonstrating the number of genes in different neutrophil subpopulations.**(I)** AUCELL quantifies NET activity in different neutrophil subpopulations.**(J)** Violin plot demonstrating NETs scores in different neutrophil subpopulations.**(K)** Bar graph demonstrating the results of enrichment analysis of different neutrophil subpopulations.

To investigate the status of infiltrating neutrophils in both heart failure and normal cardiac tissues, we conducted a comprehensive search within the GEO database. Subsequently, we acquired the GSE122930 dataset, which comprises 7 mouse hearts. This dataset includes two samples at 1 week post-TAC, two at 4 weeks post-TAC, one at 1 week post-SHAM, and two at 4 weeks post-SHAM, aligning with the objectives of our study. After rigorous quality control and meticulous screening, we identified a total of 10 distinct cell subpopulations from an initial pool of 17,918 cells, which were subsequently subjected to downstream analysis (Figures 2A, D). Importantly, when compared to the sham-operated mice, our cell scale plots revealed a substantial increase in neutrophil infiltration in mice observed 4 weeks after TAC (Figure 2C). Subsequently, the two neutrophil-related subpopulations (Neutro/Mono and Neutrophil) were extracted individually. Unsupervised clustering was then performed again, identifying three distinct cell clusters: Neutro/Mono (638 cells), Neutrophil 1 (409 cells), and Neutrophil 2 (404 cells) (Figures 2E,G). Additionally, the violin plot illustrates the number of genes expressed within these three cell subpopulations, with Neutro/Mono exhibiting the highest gene expression and Neutrophil 1 showing the lowest (Figure 2H). To gain insights into the diffrences between these neutrophil subpopulations, we quantified the activity of the NETs pathway using the AUCELL method, revealing that Neutrophil 1 displayed significantly higher activity in NETs-related gene sets in comparison to Neutrophil 2 and Neutro/Mono (Figures 2I, J). Furthermore, the results of our enrichment analysis shed light on the distinct functions of these three neutrophil-associated subpopulations (Figure 2K). Specifically, Neutro/Mono appeared to be linked with myeloid cell differentiation, Neutrophil 1 exhibited associations with the regulation of neutrophil chemotaxis, and Neutrophil 2 displayed a role in the positive regulation of leukocyte differentiation. Notably, all three cell subpopulations exhibited enrichment for terms related to T cell activation. Additionally, we conducted an assessment of the overall distribution of cell populations within both normal and disease groups within the dataset, employing UMAP plots, and provided characterization for each cell population utilizing known cell markers (Figures 2B, F).

### Trajectory analysis of neutrophils

**FIGURE 3:**
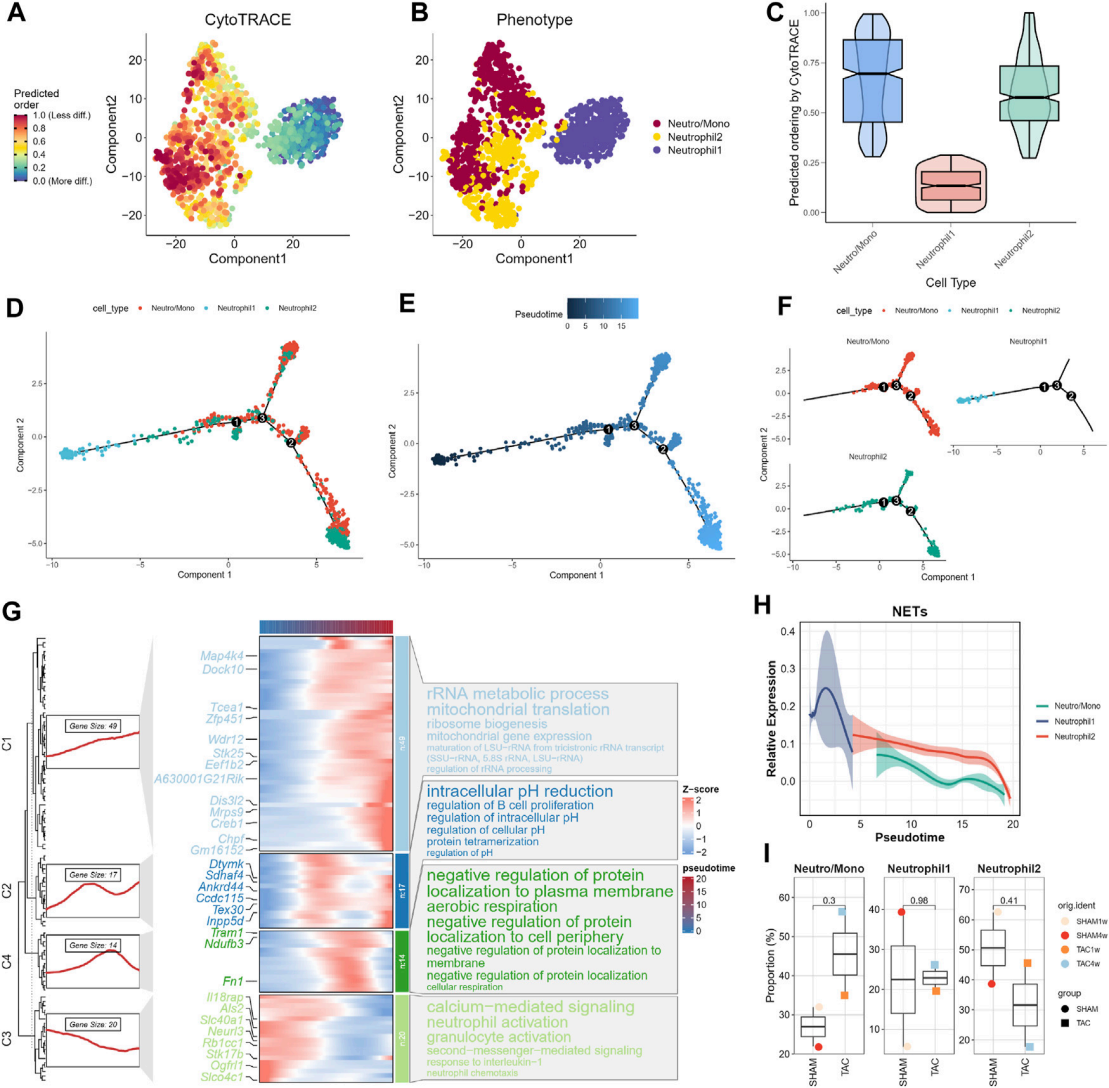
Trajectory analysis of neutrophils. **(A–C)** CytoTRACE predicts the cell differentiation potential of neutrophil subpopulations. **(D)** Distribution status of different neutrophil subpopulations in pseudotrajectory analysis. **(E)** Overall trajectory analysis of neutrophil subpopulations. **(F)** Independent distribution status of different neutrophil subpopulations in pseudotrajectories. **(G)** Distribution of key genes in trajectories and heatmap of gene enrichment analysis at different stages. **(H)** Dynamic expression profile of NETs activity. **(I)** Cell proportions of neutrophil subpopulations in different experimental subgroups.

We assessed the differentiation capacity of neutrophil-associated cell subpopulations using CytoTRACE. Neutro/Mono exhibited the highest predicted differentiation potential, while Neutrophil1 was predicted to have the lowest differentiation capacity (Figures 3A–C; Supplementary Table S3). This suggests that the Neutro/Mono cell subpopulation may play a role in initiating the differentiation of the neutrophil population. To further elucidate these findings, we integrated this result with pseudotrajectory analysis. Our analysis revealed that Neutrophil1 was positioned at the end of the differentiation trajectory, Neutrophil2 was located at the pre-mid differentiation stage, and Neutro/Mono was situated at the pre-differentiation stage (Figures 3D–F). This suggests that Neutrophil1 may represent mature neutrophils, whereas Neutrophil2 represents immature neutrophils. We also generated a heatmap illustrating the key genes involved at each stage of the neutrophil differentiation process, along with the results of enrichment analysis at different stages (Figure 3G). Additionally, curve graphs were used to visualize the NETs activities of different cell subpopulations along the pseudotemporal ordering, with Neutrophil1 exhibiting the highest activity (Figure 3H).

Finally, we combined the results of the pseudotrajectory analysis to demonstrate changes in the proportions of different neutrophil-associated subpopulations in various experimental subgroups (Figure 3I). Given that one mouse heart sample was lost due to sample wetting failure 1 week after SHAM surgery in the original study, resulting in a smaller number of cardiac tissues in the SHAM group compared to the TAC group, we focused on the changes in cell proportions in TAC1w and TAC4w. This period represents a critical transition from cardiac hypertrophy to heart failure. During this period, the proportion of Neutrophil1 cells slightly increased, while the Neutro/Mono cell proportion significantly increased. This suggests that bone marrow hematopoiesis remained active during this time. In contrast, the proportion of Neutrophil2 cells significantly decreased, indicating that this period allows for the differentiation of more young neutrophils into mature neutrophils.

### Cell communication analysis

**FIGURE 4:**
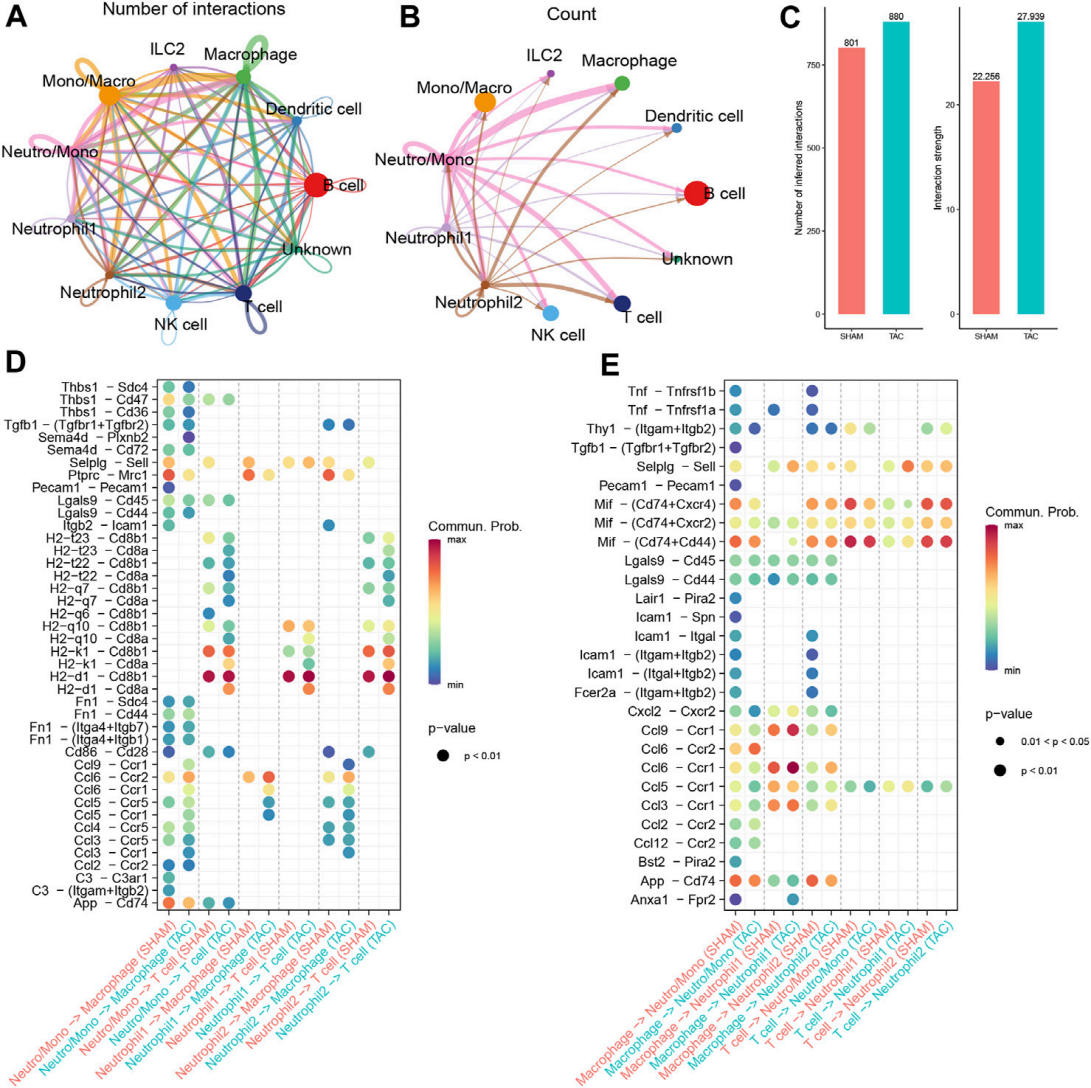
Cell Communication Analysis. **(A)** The number of interactions among immune cell subpopulations in the hearts of TAC mice. **(B)** Communication condition between neutrophils and other cell clusters. **(C)** Number and strength of interactions between SHAM group and TAC group. **(D–E)** Receptor-ligand pairs of different neutrophil subsets interacting with macrophages and T cells.

We performed CellChat analysis to identify key cell subpopulations and receptor-ligand pairs involved in interactions with neutrophils. Initially, we explored the communication patterns among all immune cell subpopulations and their interactions with neutrophils in TAC mice. Our findings indicated that macrophages were the most active communicating cell subpopulation in TAC mice (Figure 4A). Both macrophages and T cells displayed close communication with neutrophil-associated clusters (Figure 4B). Furthermore, the overall number of immune cell subpopulations and the strength of intercellular communication were increased in TAC mice compared to the sham-operated mouse group (Figure 4C).

To gain insights into the specific signaling pathways associated with neutrophils, we examined the communication between neutrophil-associated subpopulations and other cellular subpopulations, considering neutrophils as receptors and senders, respectively. We focused on understanding the communication between neutrophils and macrophages, taking into account cell subpopulations and experimental groupings. Combining these results with our trajectory analysis, we observed a progressive increase in chemokine expression in neutrophils during maturation, particularly in the hearts of mice in the TAC group (Figure 4D). Previous reports in the literature have indicated that neutrophils are the first immune cells recruited in large numbers into the myocardium after pressure overload. They produce cytokines and chemokines to attract splenic-derived macrophages to migrate into cardiac tissues. Consequently, we paid particular attention to the communication with macrophages when neutrophil 1 served as a sender. Our findings revealed that the Ccl6-Ccr2 receptor-ligand pair exhibited the closest communication. Studies show resident CCR2+ cardiac macrophages promote neutrophil infiltration into injured myocardial tissue (Li et al., 2016).

Moreover, we also focused on the communication between neutrophils and T cells. The results of our previous enrichment analysis suggested that all three types of neutrophil-associated subpopulations might be related to T cell activation. We observed a significant number of H2-Cd8 receptor-ligand pairs in the communication signals when neutrophils served as senders (Figure 4E). Cd8, acting as a co-stimulatory molecule, interacts with MHC I molecules to enhance TCR recognition of MHC-antigen peptide complexes (H2 molecules in mice), thereby promoting T-cell activation. However, when combined with trajectory analyses, most of the receptor-ligand pairs either disappeared or showed reduced intensity during the transition from Neutro/Mono to mature neutrophils. This implies that the ability to activate T cells may not be specific to neutrophils.

### Screening of NETs-related biomarkers in HF using machine learning

**FIGURE 5:**
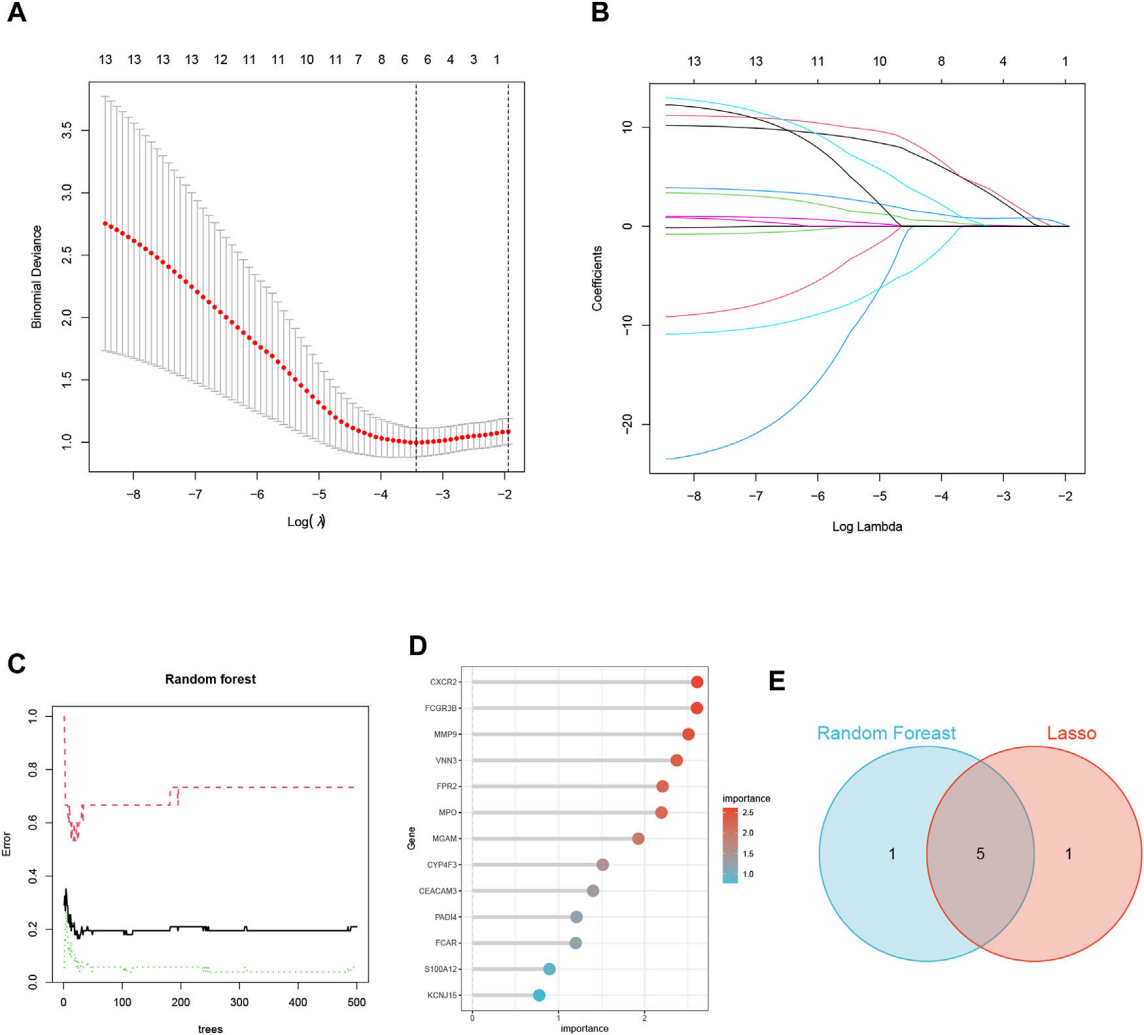
Screening of NETs-Related Biomarkers in HF Using Machine Learning. **(A)** Map of the regression coefficients of the 9 genes in LASSO model. **(B)** 6 hub genes screened by 10-fold cross-validation in the LASSO regression model. **(C)** The influence of the number of decision trees on the error rate. **(D)** The Gini Coefficient Method Achieved in Random Forest Classifier Results. **(E)** Venn Diagram Illustrating the Overlap of Two Machine Learning Screens for Genes.

In the training set GSE145154, we employed two machine learning algorithms to identify the featured genes from among the candidate key genes in heart failure patients. In the Lasso regression analysis, we inputted the 13 NETs-related genes and performed a 10-fold cross-validation (Figures 5A, B). We used lambda, determined based on the minimum binomial deviation, as the criterion, ultimately identifying five candidate genes. Additionally, we utilized the RF machine learning algorithm to rank these 13 genes based on their importance variables. Genes with a MeanDecreaseGini greater than 2 were extracted (Figures 5C, D). Through taking the intersection of genes from the LASSO and random forest algorithms, the study found five common signature genes, namely, CXCR2, FCGR3B, VNN3, FPR2, and MPO (Figures 5E; Supplementary Table S4).

### Validation of key biomarkers

**FIGURE 6:**
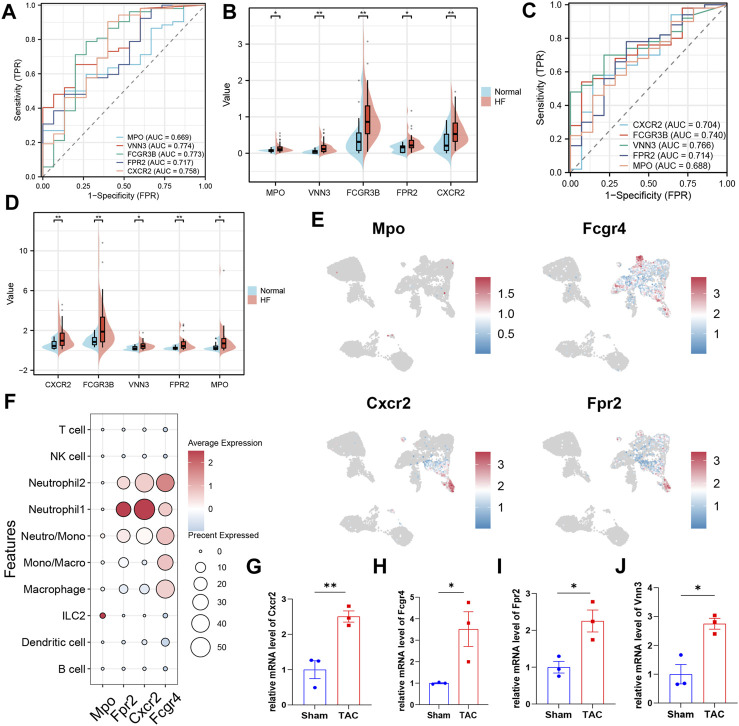
Validation of key biomarkers. **(A)** ROC curves were conducted for evaluations of the diagnostic potential of candidate genes in the GSE145154 dataset. **(B)** The pod plot showed gene expression differences between HF and normal groups in GSE145154 (a wilcoxon rank sum test was uesd). “***,” “**,” “*” represent *p* < (0.001, 0.01, 0.05).**(C)** ROC curves were conducted for evaluations of the diagnostic potential of candidate genes in the GSE116250 dataset. **(D)** The pod plot showed gene expression differences between HF and normal groups in GSE116250 (a wilcoxon rank sum test was uesd). “***,” “**,” “*” represent *p* < (0.001, 0.01, 0.05). **(E)** The UMAP plot illustrates the distribution of key genes across subpopulations of immune cells in the mouse heart. **(F)**The dot plots displayed the expression of key genes across various subpopulations of immune cells. **(G–J)** qRT-PCR to verify the expression of key genes (3 mice per group, a Student’s t-test was used). “***,” “**,” “*” represent *p* < (0.001, 0.01, 0.05).

In this study, we utilized subject working characteristic curves to assess the diagnostic value of five key biomarkers in HF. Our results indicate that HF patients had elevated expression levels of all key genes (Figures 6B, D). In the training set GSE145154, all pivotal genes showed an AUC greater than 0.700, excluding MPO, and VNN3 had the highest diagnostic value with an AUC of 0.774 (Figure 6A; Supplementary Table S5). In the validation set GSE116250, the diagnostic value of the identified key genes was further confirmed, all of the key genes demonstrated significant diagnostic value except for Mpo, which had an AUC greater than 0.700 (Figure 6C; Supplementary Table S6). Lastly, we analyzed the expression of these biomarkers in mouse cardiac immune cells. We found that while VNN3 was undetectable in the immune cell population, the other four genes were expressed (Figure 6E). Among these genes, Mpo exhibited predominant expression in ILC2 cells, whereas Fpr2 and Cxcr2 displayed higher expression levels in neutrophil 1 compared to other cell types. Fcgr4, on the other hand, was expressed in neutrophils, monocytes, and macrophages, with slightly higher expression in Neutrophil2 compared to other cells (Figure 6F). To validate these findings, we examined the expression of these five genes in both TAC mice and SHAM mice. The results revealed that Cxcr2, Fpr2, and Vnn3 exhibited significantly higher expression in TAC mice compared to the control group (Figures 6G, I, J). However, Mpo expression was too low to be reliably quantified, and Fcgr4 did not show any significant differences between the groups (Figure 6H; Supplementary Table S7).

## Discussion

The treatment of HF is a significant challenge for experts and researchers, given its complex pathogenesis and irreversible nature (Wang et al., 2017). Historically, innovative drugs such as ACE inhibitors have been the primary method of treating HF. However, recent studies suggest that immune cells, particularly neutrophils, may play a role in the disease’s progression. In mice with ANGII-induced HF, DNaseI administration resulted in the clearance of NETs and reduced cardiomyocyte death (Tang et al., 2022). Furthermore, neutrophil depletion was shown to reduce TAC-induced hypertrophy and inflammation, thus preserving cardiac function (Wang et al., 2019). While there is some direct experimental evidence implicating NETs in the progression of HF disease, our current understanding of this aspect remains limited, highlighting the need for further research in this area.

This study employed a comprehensive analysis by combining single-cell sequencing and bulk transcriptome sequencing to elucidate the biological significance of NETs in HF from multiple perspectives. Using two machine learning algorithms, LASSO and random forest, the study identified five NET-related biomarkers in HF patients: CXCR2, FCGR3B, VNN3, FPR2, and MPO, and experimental verification confirmed that the expression of the first four of these genes was significantly elevated in heart failure mice.

Among these biomarkers,FcγRIIIb is a glycosphingolipid (GPI)-anchored receptor exclusively expressed on neutrophils and plays a crucial role in the activation of NETs released by neutrophils (David et al., 2005). Previous studies have reported that the kinases Syk and TAK1 are involved in the signaling pathway that leads to the formation of NETs upon FcγRIIIb stimulation in neutrophils (Fonseca et al., 2018). CXCR2 is a prominent chemokine receptor expressed on neutrophils. Studies have demonstrated that CXCR2 and its downstream pathways through IL8 agonism mediate the classical pathway of NETosis (Chen et al., 2021). Moreover, the CXCL1-CXCR2 axis mediates cardiac hypertrophy and remodeling in HF model mice by regulating monocyte infiltration (Wang et al., 2018). FPR2 is a multifunctional G protein-coupled receptor with a 7-transmembrane structural domain (Dahlgren et al., 2020). Previous studies have reported that FPR2 can reduce hyperosmolarity-induced NETosis, which helps alleviate dry eye (Tibrewal et al., 2014). In acute HF, Fpr2 triggers increased infiltration of immature and inactive neutrophils in the heart (Kain et al., 2019). VNN3 is a secreted and membrane-bound exoenzyme involved in the conversion of pantothenic acid and cysteamine. A previous study utilizing blood transcriptome-based molecular signatures identified VNN3 as a potential diagnostic biomarker for ST-segment elevation myocardial infarction. Out of the five NET-related genes, we discovered that CXCR2, FPR2, and FCGR4 demonstrated significantly higher expression in Neutrophil 1 as compared to Neutrophil 2. This finding corroborates our observations in the mouse single-cell dataset.

The single-cell sequencing data used in this study were derived from previous studies, but our study differs from the original analysis. The original study classified neutrophils into two classes of cells by unsupervised clustering and identified CXCR2 as a gene specifically expressed in Neutrophil1, but failed to further discuss the function and significance of neutrophils. In our present study, we harnessed advanced cellular annotation tools to achieve precise annotation of key cellular subpopulations. Subsequently, we employed various methods to uncover distinct functions within neutrophil subpopulations, delve into the intricacies of neutrophil differentiation, and identify pivotal receptor-ligand pairs regulating intercellular interactions. These findings significantly contribute to our understanding of neutrophils in the context of heart failure.

The present study is subject to limitations, one of which is the relatively low number of neutrophils obtained from single-cell sequencing data. This could be attributed to several factors, such as the fact that neutrophils are not resident in cardiac tissue and only migrate to sites of injury or inflammation to perform their functions. Moreover, neutrophils have lower levels of gene expression and are more sensitive to the experimental environment, which may affect their ability to be captured by single-cell sequencing methods. It is challenging to obtain a sufficient population of neutrophils in single-cell sequencing studies without specifically targeting this cell type. Therefore, future experimental studies are still required to confirm our findings.

## Conclusion

In this study, we conducted an in-depth investigation into the functions of neutrophil subpopulations that infiltrate cardiac tissue in TAC mice. Additionally, we identified four biomarkers (CXCR2, FCGR3B, VNN3, and FPR2)associated with NETs in HF. Our findings enhance the understanding of immunology in HF.

## Data Availability

The original contributions presented in the study are included in the article/Supplementary Material, further inquiries can be directed to the corresponding author.

## Supplementary material

Supplementary Table S1 69 initial biomarkers of NETs.

Supplementary Table S2 13 NETs-related differential genes were obtained between HF and control samples.

Supplementary Table S3 The CytoTRACE values of two clusters of neutrophils.

Supplementary Table S4 Biomarkers related to NETs identified through machine learning.

Supplementary Table S5 The expression status of five NETs-related biomarkers in the GSE145154 dataset.

Supplementary Table S6 The expression status of five NETs-related biomarkers in the GSE116250 dataset.

Supplementary Table S7 Relative expression of four key genes in the SHAM and TAC groups.
